# A set of web-based public decision support tools for integrated planning and management in aquaculture

**DOI:** 10.1016/j.mex.2022.101795

**Published:** 2022-07-25

**Authors:** Junyong You, Liangju Yu, Julien Meillon, Aline Gangnery, Cedric Bacher, Hui Liu, Øivind Strand

**Affiliations:** a NORCE Norwegian Research Centre AS, Nygårdstangen, Bergen 5838, Norway; b Dalian Ocean University, Dalian 116086, China; c Ifremer, SISMER, Plouzané F-29280, France; d Ifremer, DYNECO, Plouzané F-29280, France; e Yellow Sea Fisheries Research Institute, Chinese Academy of Fishery Sciences, Qingdao 266071, China; f Institute of Marine Research, PO Box 1870 Nordnes, Bergen 5817, Norway

**Keywords:** Marine spatial planning, Web Map Service (WMS), Geographic Information System (GIS), Aquaculture site selection, Suitability assessment

## Abstract

We developed a set of web-based tools to meet the demand for spatial planning and help to determine the available space suitable for marine aquaculture activity. These tools were derived from AkvaVis concept, which was initially designed for the management of Norwegian aquaculture. The AkvaVis concept was adapted to different national aquaculture contexts and two other tools were developed in France and China. Besides using GIS maps and thematic layers, interactive functions were added to enable the user to select spatial parameters, build indicators for aquaculture siting and instantly display the requested information. For each tool, we describe the main technical features, input data, data geoprocessing, output products, tool strengths and limits, and applicability to other case studies.

The three tools we present share common concepts and features:

• use of standardized protocols for data (Web Feature Services, Web Map Services)
• reusability of the modules developed for applications to other case studies
• web-based interface for spatial data viewing and processing

They also show some differences, e.g., the Chinese tool exists as a desktop or a web-based support system. Differences and demonstrations for different aquaculture contexts in Europe and China offer some flexibility in future applications.

## Graphical abstract

**Figure:**
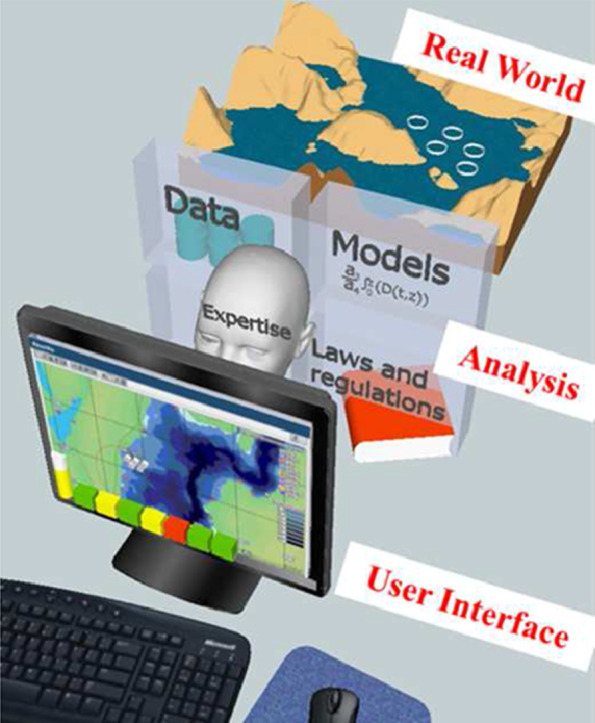

Specifications table

**Table:** 

Subject areas:	Decision support system, software development
More specific subject area:	Marine spatial planning, Web Map Service, aquaculture management
Method name:	AkvaVis decision support tool
Name and reference of original method:	Web-based public decision support tool for integrated planning and management in aquaculture, https://doi.org/10.1016/j.ocecoaman.2020.105447
Resource availability:	AkvaVis: http://akvavis.no/ SISAQUA: https://wwz.ifremer.fr/sisaqua/

**Method details**

## Introduction

Implementation of spatial planning and management processes in aquaculture require efficient, effective and useful decision support tools for making information accessible to users and stakeholders. Such information may have varied scales (individual, local, regional, global) and dimensions (physical, ecological, economic, social/cultural), and importance in decision making. In the companion paper [1], we have developed several decision support tools based on a common concept of AkvaVis for integrated planning and management in aquaculture. The AkvaVis concept was initially designed as part of a comprehensive aquaculture management approach developed in Norwegian aquaculture, where demand was identified for improving accessibility and integration of environmental and regulatory information in site selection, spatial planning and licensing [2]. The tool uses GIS maps and thematic layers with the addition of an interactive function where choices relating to spatial parameters can be made by the user and the tool instantly displays the requested information. Tool versions for demonstration included a dynamic interface between the user and an integrated system of data compilation, model simulations, coastal zone development plans, regulatory framework analysis and assessments [2,3]. The AkvaVis concept was adapted and developed for use within four case studies which deal with very different scales of aquaculture, management frameworks and issues related to aquaculture in four different countries [4]. These cases applied specific methods and technical features to adapt the concept, resulting in the developments of the Spatial Information System for AQUAculture (SISAQUA) in France, the Aquaculture Planning Decision Support System (APDSS) in China, while AkvaVis was readily applied in Norway and Northern-Ireland. Here we complete the companion paper [1] by providing the methods and technical information of these adapted tools, their benefits and limitations including reproducibility and applications to other areas.

## AkvaVis specifications

### Tool technical features

AkvaVis was developed in collaboration between Institute of Marine Research and Hordaland County Council for conceptualizing related to management and providing models and data, and Christian Michelsen Research developing the technical features. Based on the Web Map Service (WMS) technology, AkvaVis implements several functionalities, e.g., visualization of environmental data (current, wave, depth, etc.), existing aquaculture facilities, source farm locations and dispersion of parasites (salmon lice), and virtual siting of aquaculture farms.

**Fig. 1:**
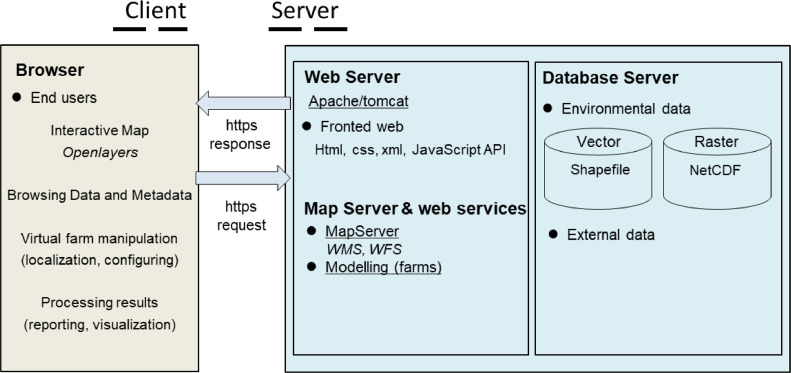
Graphical representation of AkvaVis.

AkvaVis contains two main parts: “client” for interface to attain user requests and displaying results upon requests, and “server” for implementing respective functionalities. AkvaVis client was developed by HTML techniques (JavaScript, CSS) and Adobe Flash (Action script), and the server was developed by Java following the standard MVC (Model, View, Controller) structure (Fig. 1). The main development has been focused on the server part. Different modules are implemented, including WMS (CRS: coordinate reference system, WFS: web feature service, map manipulation), farm module for controlling a virtual farm, aquaculture models for running relevant computational models for different species, and data management module to handle the relevant environmental data. The AkvaVis software package was packed into a WAR file, which is then deployed in Tomcat. Certain functionalities can be accessed via APIs.

AkvaVis codes were stored in an internal repository, and advanced developed tools, e.g., Intellij IDEA Ultimate as IDE, Gradle for project management, Subvision for version control, have been used when developing the tool by a development team.

AkvaVis is running on an internal server with irregular checks of running logs. The codes are not public, while the service is open to the public. AkvaVis development was supported by national funding and EU Horizon 2020 funding.

### Input data

AkvaVis mainly uses raster data in the Netcdf format.

AkvaVis layer presentation is based on locally stored data. It can also receive and display external data as additional layers, given that the external data sources follow the common Web Map Service (WMS) protocol.

In principle, the data should follow standardized protocols defined by the Open Geospatial Consortium (OGC), e.g., WMS for layer visualization, map manipulation, and Web Feature Service (WFS) for data/layer meta information display. In addition, AkvaVis does not produce data, its main functionality is to display data on an electronic map and make decision support.

The nature of data integrated to each tool in relation with the different case studies are extensively described in the Appendix 3 of the companion paper [1] (https://ars.els-cdn.com/content/image/1-s2.0-S0964569120303549-mmc3.docx). Bathymetry, currents, shoreline are typical data required for aquaculture spatial planning. Data related to socio-economic (e.g. location of sewage outlets) and governance and regulations (location of already existing aquaculture sites or conservation areas) are also relevant.

### Geoprocessing and output products

WMS and WFS techniques were employed to convert the Netcdf data to layers with geographical coordinates, meta information about individual data can also be displayed on the map. AkvaVis generates geographical layers based on the supplied data, and a spatial suitability report is also generated based on the suitability analysis of a chosen location of aquaculture site.

**Fig. 2:**
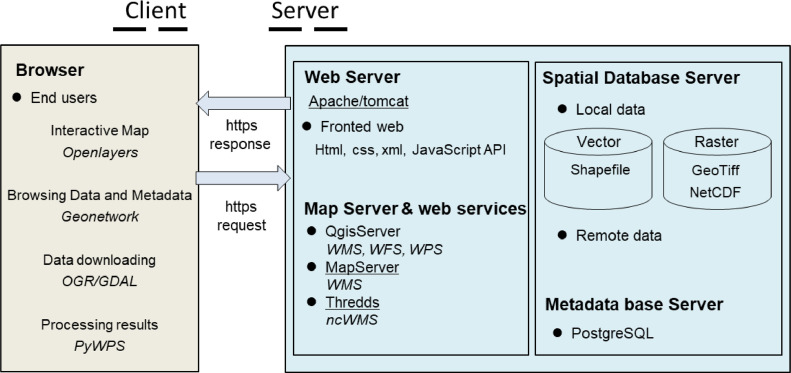
Graphical representation of SISAQUA.

**Fig. 3:**
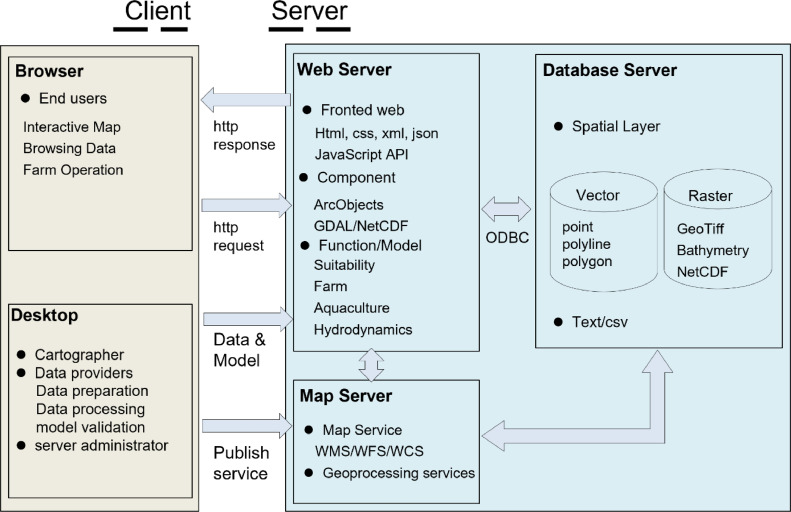
Graphical representation of APDSS.

Panel screenshots showing output examples for each tool and case study are shown in Figs. 2 to 5 in the companion paper [1].

### Tool benefits and limits

A limitation experienced in AkvaVis is episodic instability under high requests, and the background map of AkvaVis is dependent on external WMS services. On the other hand, AkvaVis is a daughter product from a family of software packages under the same WMS module. A common module has been developed for WMS serving, and individual clients can be easily and agilely implemented. AkvaVis has provided a simple user manual.

### Reproducibility and application in other cases

AkvaVis is developed following the protocol of WMS, and many fundamental modules have been implemented. Therefore, these modules can be easily reused in many other map applications. There are several other tools developed by Christian Michelsen Research that share the common WMS modules, e.g., a tool for AIS (Automatic Identification System) signal processing.

On the other hand, the architecture of AkvaVis tool has been elaborately designed such that the tool can be directly extended to other aquaculture areas. For example, if only the functionality of spatial planning for new aquaculture farms is needed, AkvaVis can directly be used in other areas given that the relevant environmental data is provided. This was also the main reason why the tools SISAQUA and APDSS have heavily adapted the concept of AkvaVis, with application in four different areas, environments, and regulatory frameworks. By only minor changes AkvaVis has performed when adapted to new areas.

## SISAQUA specifications

### Tool technical features

SISAQUA is a web-based tool relying on the Spatial Data Infrastructure Sextant (SDI, https://sextant.ifremer.fr/). This SDI has been developed by Ifremer over 20 years evolving with technology: norms updating, new standards, and new software. The tool is based on a set of free software to which Sextant also contributes. Main architecture relies on a metadata catalogue describing the data through the Geonetwork software (version 3.10; Fig. 2). The language used is XML. Metadata really constitute the core of the tool and guarantees that all data access services (viewing, downloading, processing or even filters) are associated to metadata. In addition SISAQUA offers a map viewer relying on the Openlayers solution. Data are displayed through Web Map Services (WMS), which are implemented using the software Qgis Server (version 3.16), Mapserver (version 7.6) and Thredds (version 4.6). Data downloading is achieved by direct access (e.g. ftp) or through a Geospatial Data Abstraction (GDAL/OGR) library. This library allows choosing of the format and the projection when downloading the data. An overlayer is added to these softwares to customize the display of the tool. The tool is finally deployed as an Application Programming Interface (API) that can be completely customized. Javascript is the language of this API.

SISAQUA is managed with respect to Sextant architecture, which relies on three pillars and specific Ifremer services: administration, development, and maintenance under operational condition. Global funding of Sextant is based on a perennial own funding warranting the durability of the tool as well as the assimilation of the continuous evolution of computer science technology. Sextant also benefits from occasional funding from regional, national or European thematic projects. For instance, indicators produced by online treatments and proposed by SISAQUA did not exist in the previous version of Sextant and were added specifically with the financial help of the AquaSpace European project. The process follows three different steps: (i) agreement on the principle of adding a specific functionality, (ii) development of the functionality and (iii) implementation in Sextant. These treatments finally benefit all Sextant users.

### Input data

SISAQUA offers access to various data like vector data represented by points, lines, or polygons as well as raster data (images). They are either stored and saved locally on Ifremer's own servers or harvested from other devices through remote services (e.g. WMS).

Data management/administration is based on a set of norms and standards widely used by other web-based mapping tools. The description of the data (metadata) follows the European Directive INSPIRE as well as the ISO19115 and ISO19139 standards. Regarding data access, the tool relies on the standardized protocols of the Open Geospatial Consortium (OGC) such as the WMS for visualization, the Web Coverage Service (WCS), WFS for downloading and the standard Web Processing Service (WPS) for online processing. Locally, administration rights of the tool are attributed to a list of users managing the website, data catalogue and web services as well as the API.

### Geoprocessing and output products

Geoprocessing for computing SISAQUA indicators, is made through a linkage between Qgis software and PyWPS, which is an implementation of the WPS standard. PyWPS is written in Python. In the case of SISAQUA, original treatments implemented in Sextant for one layer were adapted to manage several layers at the same time and then combine several data. The dialog box for the treatment can be customized in terms of titles, addition of help, input locking, or default values. The output product is a map that can be displayed directly in the viewer but also downloaded. The tool is upgradeable and could integrate other treatments or other outputs (graphs, reports, etc.).

### Tool benefits and limits

A key strength of SISAQUA is that it relies on Sextant SDI, which ensures a continuous maintenance under operational condition. Presently, about thirty other data access portals use the Sextant API. Among these portals, one finds the Odatis ocean Pole giving access to oceanographic data and mainly designed for the French scientific community (https://www.odatis-ocean.fr/); the Information System for the Marine Environment gathering various public information and depending on the French state (https://www.milieumarinfrance.fr); the JERICO Research Infrastructure representing the European gateway for coastal observation (https://www.jerico-ri.eu/). Sextant is described through numerous guides (in French), available on the Sextant website (https://sextant.ifremer.fr/eng/Ressources/Guides-and-supports).

At this stage, there are still ways to improve SISAQUA, such as the implementation of metadata on treatments themselves. The biggest limitation concerns the access to external services. Regular monitoring is required to ensure that remote data are still being served by the data providers. This type of problem has been noted and corrected on several occasions since the implementation of the tool. There is also space for the development of indicators and to generate automatic reports to help decision-making.

### Reproducibility and application in other cases

SISAQUA is based on a map viewer which makes available approximately 120 local, regional or national data sources. To reproduce the SISAQUA application in another geographical area or with a slightly different aquaculture issue, the main work would consist of a complete inventory of the data to be published online. Data should comply to data standards (WMS, WFS, CSW), whatever the source of data - e.g. data produced by Ifremer or distributed by another organization. As for SISAQUA, it would be possible to integrate data created specifically for this new application by writing a metadata and creating the associated distribution services.

Besides, the tool would require very few adjustments:

- the technical base would be the same and only a few minor configurations would need to be made (XML for the map context, API code to be deployed in a website)

- the metadata catalog should be filled in to provide a standardized description of each data

## APDSS specifications

### Tool technical features

APDSS is an ArcGIS-based spatial planning tool (system), which adopts C/S (Client/Server) + B/S (Browser/Server) mixed mode for development, by giving full play to the advantages of the two modes. The tool has also applied a set of free software that ArcGIS is compatible with, such as Python. This tool contains two parts including a desktop part and a web-based part, which have differentiated functions (Fig. 3). The desktop system provides data storage, updating and processing, model simulation and analyses; while both systems can accomplish a range of tasks, such as, data query and browsing, display of model simulated results and map layers, and map retrieval.

(1) Architecture of the desktop-based APDSS: The map viewer of the desktop system adopts user-friendly WinForms embedded with ArcEngine Object components, such as Map Control component and TOC (Tool of Control) component. All these functions are developed using C^#^ language in the .NET Framework environment.
(2) Architecture of the web-based APDSS: The map viewer is running based on ArcGIS API for JavaScript and ArcGIS Server, where the base map layer is loaded by satellite raster tiles from Esri ArcGIS Image Server, and in contrast, the other feature layer such as raster layer or shape file are published online from local ArcGIS server. The Web-based APDSS is developed in Asp.Net-Microsoft web platform. The languages adopted in web-APDSS involved JavaScript, php, html, CSS, and PHP for server-side processing.

The system is deployed to the client's environment where actual users can run the system. All data and components are stored locally. System administrators can make necessary enhancements, corrections, and changes for maintenance to ensure the system works normally.

### Input data

There are several icons in the toolbar above the top of the map viewer of the desktop APDSS tool. Each of these icons launches a widget. Data and associated metadata in four types of formats, such as vector (shapefile), raster and NetCDF data, and non-spatial data in excel format, are imported and processed into the system by these widgets. In the APDSS map viewer, users can manage and browse data in the catalog. A pop-up is also available in the catalog pane that shows metadata (name of data, type, date, and location). In addition, environmental data storage is one of the main functions of the desktop APDSS. The input data are all stored locally before they are published on ArcGIS for server by server manager.

Afterwards, the clients of web service can receive data from the database server. For instance, the client users can access environmental data and simulated data for the mariculture waters with the web-based APDSS tool, perform data browsing, and process and display model results against different geographical layers.

### Geoprocessing and output products

The ordinary users (managers and fish farmers) can execute different geoprocessing tasks from the web server, such as browsing environmental data with WMS maps and thematic layers in map server. Other tasks may include extracting data by selecting data with certain characteristics, finding suitable locations for aquaculture, or predicting and checking the growth status.

The APDSS tools involve basic map operation, and the final output of products are raster geographical layer or vector geographical layer from non-spatial data. On one hand, it can load WMS/WFS data online from the Image server. On the other hand, many local environmental parameters of the aquaculture sea being surveyed are originally in the form of raster or point thematic layers, their proximate raster layers are transformed from NetCDF format's hydrodynamic data by GDAL and NetCDF DLL components. In contrast, the water quality parameters are extracted from field survey samples in excel format, they should be transformed to point layers, and finally interpolated into raster data in geoprocessing services tasks using python.

### Tool benefits and limits

APDSS was developed by taking full consideration of biological, environmental, physical dynamics and management perspectives of aquaculture. It offers decision support for aquaculture site selection by integrating applications of map-based dynamic environmental data display, model simulation of species growth, and aquaculture carrying capacity. This tool can also be used as a reference for fish farm management, aquaculture permit authorization, and even the zoning of aquaculture waters and drawing of marine ecological protection redline (in China's case).

However, just like any other spatial planning tools, APDSS may be limited by the availability and accuracy of environmental monitoring data. Currently the system has only a Chinese version, and an English version may be necessary if its application needs to be further extended and improved.

### Reproducibility and application in other cases

This tool is mainly designed to aid aquaculture planning decisions, and it can store and display many types of data, including vector data, raster data and NetCDF data, and sample data by some DLL component and Java's API. It is easy to reproduce the tool's function in other similar geographical areas. However, a big inventory of data will be indispensable when the tool is going to be developed for other aquaculture waters. As explained in part 4.4, the biggest challenge for the extended use of APDSS is the lack of observational data. APDSS is largely based on many year's ecological study of the aquaculture in Sanggou Bay, where the geographical, environmental, biological, and ecological characteristics have been thoroughly investigated.

Despite these challenges, APDSS application has great potential for more mariculture waters in China, as a guide and assistance for aquaculture governance. Different applications may also be developed with the APDSS architecture, including risk assessment for aquaculture, carrying capacity evaluation, etc.

In the future, further adjustments will be necessary to continuously improve the APDSS framework, to make it a relatively more complete and independent aquaculture environmental database, and a tool for aquaculture environmental interaction assessment, to assist aquaculture zoning / planning and management.

## Conclusion

This paper presents methods and technical information of a set of decision support tools for spatial planning and management in aquaculture. Under a similar conceptual framework of combining GIS, WMS, aquaculture management and other technologies, three different tools have been developed that can provide appropriate suitability indication for assessing new farming areas. Even though the three tools share common principles in aquaculture spatial planning, they have respective features adaptive to individual management frameworks and scenarios. Users can choose respective tools according to the aquaculture environments and scales, data types and sources and issues to address. Furthermore, there is a growing need for spatial planning, displaying and sharing spatial data for the blue economy. We expect the developed tools can expand its applications also beyond aquaculture.

## Declaration of Competing Interest

The authors certify that they have NO affiliations with or involvement in any organization or entity with any financial interest (such as onoraria; educational grants; participation in speakers’ bureaus; membership, employment, consultancies, stock ownership, or other equity interest; and expert testimony or patent-licensing arrangements), or non- financial interest (such as personal or professional relationships, affiliations, knowledge or beliefs) in the subject matter or materials discussed in this manuscript.

## Data Availability

The authors do not have permission to share data.
